# Using Resuscitative Endovascular Balloon Occlusion of the Aorta (REBOA) as a Rescue Strategy in Severe Postpartum Hemorrhage: A Case Report

**DOI:** 10.3390/diagnostics14171980

**Published:** 2024-09-07

**Authors:** Sophie-Kristin Brauer, Alexandre Athanasios Musy, Sophie Schneider, Fabienne Nicole Trottmann, Nina Kaderli, Christian Vetter, Daniel Surbek, Marc Schindewolf, Anna Lea Gerber, Manuela Stotz, Wolf Hautz, Jarmila A. Zdanowicz

**Affiliations:** 1 Department of Emergency Medicine, Inselspital, Bern University Hospital, University of Bern, 3010 Bern, Switzerland; sophie-kristin.brauer@extern.insel.ch (S.-K.B.); alexandre.musy@gmail.com (A.A.M.);; 2 Department of Obstetrics and Gynecology, Inselspital, Bern University Hospital, University of Bern, 3010 Bern, Switzerland; 3 Department of Obstetrics, Spital Emmental, 3400 Burgdorf, Switzerland; 4 Department of Anaesthesiology and Pain Medicine, Inselspital, Bern University Hospital, University of Bern, 3010 Bern, Switzerland; 5 Department of Angiology, Inselspital, Bern University Hospital, University of Bern, 3012 Bern, Switzerland

**Keywords:** multidisciplinary approach, postpartum hysterectomy, resuscitative endovascular balloon occlusion of the aorta, REBOA, severe postpartum hemorrhage

## Abstract

Postpartum hemorrhage (PPH) is a leading cause of maternal morbidity and mortality. Routine treatment of PPH includes uterotonics, tranexamic acid, curettage, uterine (balloon) tamponade, compression sutures, uterine artery ligation, and, if available, transcatheter arterial embolization (TAE). In cases of severe PPH refractory to standard medical and surgical management, hysterectomy is usually the ultima ratio, and is equally associated with a higher rate of complications. In addition, this sudden loss of fertility, especially in young women, can be devastating. Here, we report a case of a 29-year-old woman who suffered from severe PPH with a blood loss > 1500 mL and hemodynamic instability after delivery of her first baby at a smaller hospital. She was consequently successfully treated with resuscitative endovascular balloon occlusion of the aorta (REBOA) by first placing a balloon catheter into the infra-renal aorta and subsequent TAE after failure of all other available treatment options prior to hysterectomy. TAE has been suggested in PPH treatment to avoid hysterectomies and thus to preserve patients’ reproductive function. If hemodynamic stabilization cannot be achieved with mass transfusion, REBOA seems to be an effective rescue strategy with which to achieve hemodynamic stabilization and gain additional time for embolization. Although REBOA is already recommended in several PPH guidelines, this approach seems relatively unknown in German-speaking countries.

## 1. Introduction

Postpartum hemorrhage (PPH) is one of the leading causes of maternal morbidity worldwide, accounting for 13–30% of all maternal deaths and occurring in approximately 1–3% of all deliveries [1]. Traditionally, PPH is defined as a loss of ≥500 mL of blood after vaginal delivery or ≥1000 mL after a caesarean section (CS). While severe PPH is not universally defined, most obstetric societies propose a blood loss of at least 1000 mL in 24 h [1,2,3,4]. Irrespective of the amount of blood loss, PPH must also be suspected with any signs or symptoms of maternal hypovolemia [1]. The leading underlying causes for PPH have been described as the so-called 4 Ts: tone (uterine atony); tissue (retained placenta or clots); trauma (lacerations or uterine rupture); and thrombin (clotting-factor deficiency). Several risk factors have been identified; however, in more than 50% of women developing a PPH, no previous risk factors were present [1,2]. Studies have shown that maternal deaths due to PPH could have been prevented if standard care had been applied [1,3].

The management of PPH depends on the amount of blood loss, the woman’s clinical condition, and the suspected origin of bleeding. A coordinated, multidisciplinary approach is essential, including early communication to the patient and other specialists such as the anesthesiologist and intensive care specialists; the accurate quantification of blood loss; monitoring of vital signs; fluid replacement; and, if necessary, mass transfusion [1,5]. The suggested first-line medical treatment in the management of PPH is the use of uterotonics such as oxytocin and sulprostone, as well as tranexamic acid. Surgical treatments include dilatation and curettage to remove potential residual placenta and the insertion of an intrauterine tamponade, usually by means of intrauterine balloon devices, which may escalate to a laparotomy in order to apply compression sutures or to ligate blood vessels. Postpartum hysterectomy is usually used as a last resort. Minimally invasive endovascular procedures, when available, may include uterine artery embolization (TAE) with gel foam, particles, microspheres, or coiling, prior to, or concurrent with, hysterectomy [1].

Special consideration must also be given to severe PPH in low-resource settings, as described in the 2022 FIGO (International Federation of Gynecology and Obstetrics) recommendations on PPH. Here, aortic compression or non-pneumatic antishock garments—a lightweight lower body compression device—have been suggested [4]. In this context, the use of REBOA could also be envisioned, as described below.

Resuscitative endovascular balloon occlusion of the aorta (REBOA) has been previously shown to be an effective hemorrhage control in non-compressible abdominal and pelvic bleeding. It involves the temporary placement of an endovascular balloon in the aorta until definite treatment is carried out [6]. Access to the common femoral artery is established, followed by the advancement of a catheter within the aorta. It is placed in one of the following aortic zones before the balloon can be inflated: (1) Zone I, the descending thoracic aorta above the truncus coeliacus; (2) or Zone III, the infra-renal abdominal aorta below the renal arteries [7]. The balloon inflation leads to a reduction in the flow of the distal circulation, whereas myocardial and cerebral perfusion are increased. The time of balloon inflation must be kept as short as possible to avoid ischemic complications [7].

Previous studies have mostly assessed the use of REBOA in trauma care [6]. In 2018, a meta-analysis examined the use of REBOA for any specialty from 1900 to 2017. The most common indication for REBOA insertion was abdomino-pelvic trauma or ruptured abdominal aortic aneurysms. Other non-traumatic diagnoses are rare, and even more rare are reported cases with PPH [8]. Only five studies are described for PPH at the time of this review.

More recent studies have shown that most patients eligible for REBOA had a non-traumatic etiology of hemorrhage. In a review of all cases that required mass transfusion, the authors identified PPH as the most common indication that could have potentially benefited from a REBOA procedure [6]. In 2017, Stensaeth reported on the fluoroscopy-free use of REBOA in 36 patients for severe PPH; uterine artery embolization was performed on 17 (47%) patients and a hysterectomy on 16 (44%) patients. Six patients suffered from complications, with one needing surgical intervention after an aortic tear; further complications included thrombotic events [9]. A more recent publication has shown that, since 2017, lower complication rates, less blood product use, and fewer hysterectomies have been reported after REBOA use [10]. In March 2022, a retrospective study from Japan on 143 women who were treated by REBOA for PPH was published [11]. The authors reported 10 deaths (7%) and 23 hysterectomies (16.1%) but did not compare treatment outcomes for PPH with and without REBOA. Further studies also suggested that unnecessary hysterectomies could be avoided by using REBOA in combination with transcatheter arterial embolization [1,11]. Even if fertility seems to be reduced after TAE, it is a valuable method with which to preserve the women’s reproductive function [11].

While German-speaking PPH guidelines describe the use of REBOA as a treatment option, this technique does not seem to be very well known, and has, to our knowledge, not been previously used in Switzerland [1]. Furthermore, REBOA use is not mentioned in either the 2022 FIGO guidelines, or in the 2016 RCOG (Royal College of Obstetricians and Gynaecologists) guidelines [3,4]. In the following article, we present the case of a patient with severe PPH and hemodynamic instability in spite of a mass transfusion protocol, where a hysterectomy was avoided by successfully using REBOA and TAE, and a full recovery was achieved.

## 2. Case Presentation

A 29-year-old woman (gravida 2, para 0) with no relevant medical history except prior dilatation and curettage due to an anembryonic pregnancy underwent a CS at a secondary care center (SCC) after two hours of failure to progress in labor at full dilatation. She delivered a healthy baby. After the CS, the woman had a physiological uterine tone and physiological blood loss. Twenty-five minutes after the operation, relevant vaginal bleeding of about 1500 mL occurred. There was no evidence of residual placental material and no relevant amount of free fluid in the abdomen, as seen by ultrasound. Misoprostol was administered to achieve uterine contraction, intravenous tranexamic acid was given additionally, and the decision for a surgical revision was made. Intraoperatively, a hemorrhage was located near the uterotomy, which was initially controlled by renewing the uterine stitches. In addition, the uterotomy was covered by TachoSil^®^ (Corza Medical GmbH, Zürich, Switzerland), a hemostatic matrix. There was no significant amount of free fluid intra-abdominally. However, the woman became increasingly hemodynamically instable. A curettage was carried out, followed by the application of compression sutures. A hemostatic intrauterine gauze (Celox™ PPH gauze, Medtrade Products Ltd., Crewe, UK) was then applied. In addition, carbetocin was given intravenously. Because of persistent vaginal bleeding, the Celox™ gauze was removed and an intrauterine balloon tamponade device (Bakri^®^, COOK MEDICAL LLC, Bloomington, IN, USA) was inserted. Then, a new Celox™ gauze was inserted into the uterus. The patient’s total estimated blood loss up to that point was >5000 mL, and the platelet blood count dropped to 39 G/L. During treatment at the SCC, a mass transfusion was undertaken by administering 13 units of packed red blood cells, 10 units of fresh frozen plasmas, 6 g of fibrinogen, 9000 mL Ringer’s lactate solution, 500 mL Voluven^®^ (Hydroxyethyl starch, Fresenius Kabi AG, Kriens, Switzerland), 2 g calcium, 1000 IE Beriplex^®^ (Factor II, VII, IX, X, Protein C; CSL Behring AG, Bern, Switzerland), 3 g tranexamic acid, and a prophylactic antibiotic therapy with cefuroxime was commenced. Due to persistent hemodynamic instability, the patient was then transferred to a tertiary care center (TCC) for further treatment. 

**Figure 1:**
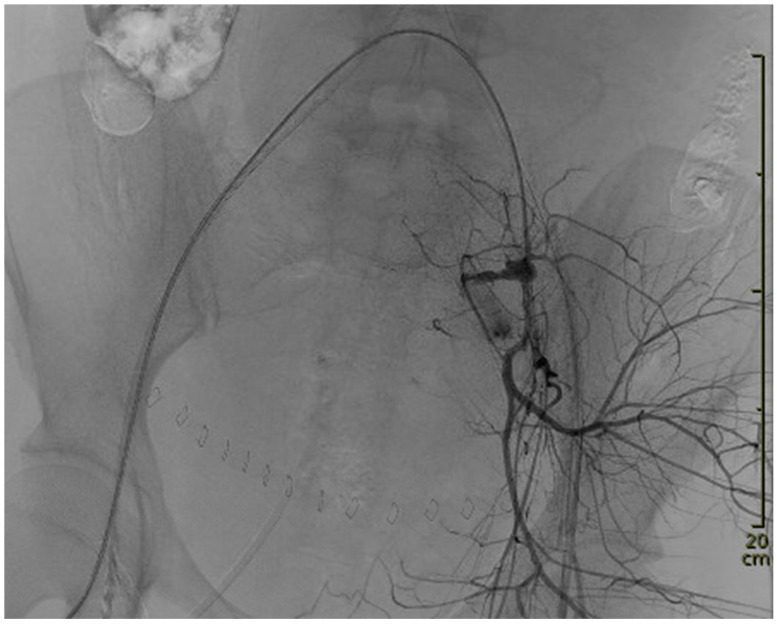
Initial angiography showing the extent of the bleeding with the REBOA in place.

Upon arrival at the TCC, the patient was intubated, hypotensive at around 90 mmHg systolic pressure, and under continuous noradrenaline infusion at 0.15 µg/kg/min. In order to guarantee a multidisciplinary approach, the following specialties were present in the TCC upon arrival or had already been informed: obstetricians; emergency care physicians; anesthesiologists; and angiologists. Mass transfusion and advanced coagulation management, including viscoelastic testing and factor supplementation, was continued. Concurrently, a 7 French sheath was placed, under ultrasound guidance, into the right common femoral artery and a REBOA catheter (ER-REBOA^®^ by Prytime Medical Inc., Boerne, TX, USA) was advanced into aortic zone III (infra-renal abdominal aorta). Less than five minutes after the decision was made to deploy a REBOA, the catheter was in place. Balloon placement was based on anatomical landmarks; the balloon was then inflated with 6 mL saline solution. After aortic occlusion, blood pressure increased, the heart rate decreased markedly, and noradrenaline was stopped within a minute. The patient was then transferred to the adjacent angiography room. The initial visualization of the bleeding is shown in Figure 1.

**Figure 2:**
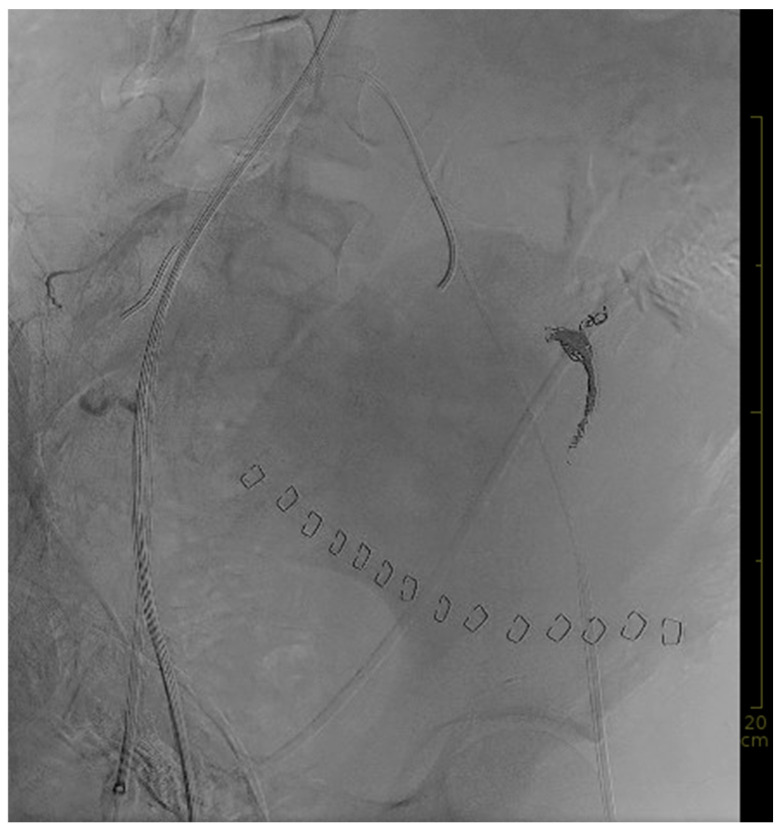
Final angiography after embolization.

Bleeding localization and embolization from a super-selective position in the left uterine artery were started within seven minutes after the angiography had commenced. For bleeding localization, the REBOA balloon was unblocked intermittently. The initial bleeding control at the site of laceration was achieved using detachable coils. To prevent sustained bleeding from possibly injured smaller vessels (not visible on the angiogram) and from the contralateral side, and to ensure vessel occlusion in a patient in severe hemorrhagic shock and consecutive deficiency coagulopathy, microspheres and gelfoam were applied in addition. Bleeding control was finally achieved using eight detachable Concerto™ coils (Medtronic, Minneapolis, MN, USA), Embozene™ Microspheres for Embolization (700 µm) (Boston Scientific, Marlborough, MA, USA), and gel foam. Hemostasis was achieved through the successful embolization of both uterine arteries, as shown in Figure 2.

Thereafter, the hemodynamically stable woman was transferred to the intensive care unit for further observation, with a continuous uterotonic infusion and antibiotic prophylaxis. The approximate duration of the intermittent aortic occlusion was approximately 70–75 min. Forty-three minutes after the REBOA balloon placement, and within seven minutes after the start of the angiography, the site of bleeding was localized, and embolization had begun. Perfusion was allowed intermittently by deblocking the REBOA balloon after initial bleeding control for diagnostic angiographic imaging and verification of hemostasis. There was no standardized inflation–deflation regimen of the balloon at the end of the procedure. The regimen was determined instead based on bleeding control and clinical stabilization of the patient. The final deblocking of the REBOA balloon was conducted slowly, over five minutes, and under the control of clinical parameters and angiographic signs of rebleeding.

**Figure 3:**
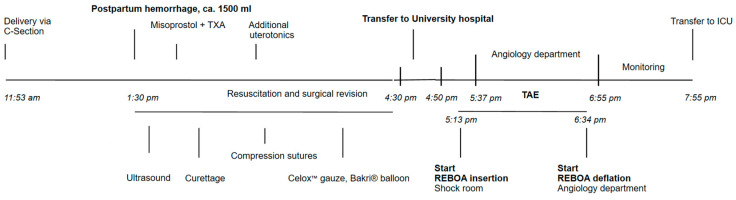
Timeline of events from onset of bleeding. ICU intensive care unit; REBOA resuscitative endovascular balloon occlusion of the aorta; TAE transcatheter arterial embolization; TXA tranexamic acid.

Within 24 h, the patient developed an anuric acute kidney injury (AKI III). Sonographically, the bladder could not be visualized with the Bakri^®^ balloon in place, but a hydronephrosis grade I was seen on the right side. A pre-existing pregnancy-associated hydronephrosis is very common on the right side, although in this case it was not previously described. The Bakri^®^ balloon and Celox™ gauze were removed after approximately 24 h (in accordance with German-speaking guidelines, the Bakri^®^ balloon is routinely left in place for 24 h [1]) and the hydronephrosis was slightly regressive. A timeline of the events is presented in Figure 3.

During hospitalization, a hematologic evaluation revealed no signs of thrombotic microangiopathy or hereditary coagulopathy. Anuria remained, and the decision to start intermittent hemodialysis was taken. Diuresis returned slowly over the following days, but intermittent dialysis was continued until discharge at day 22.

## 3. Discussion

This case demonstrates the successful use of REBOA in a hemodynamically instable patient with mass transfusion due to PPH before definite treatment and ultimately stabilization with uterine artery embolization. In addition, hysterectomy was avoided; the woman’s fertility was preserved, with no known further complications to date.

REBOA is a maneuver that requires only relatively common skills and equipment, despite being a highly invasive procedure. Emergency and intensive care unit physicians, as well as anesthesiologists, are present 24/7 in almost any hospital and are all familiar with Seldinger’s technique, which requires the placement of a femoral artery sheath, typically under ultrasound guidance. The only additional piece of equipment required is the REBOA catheter itself. Although this technique is increasingly used in Swiss trauma centers [12], and, within the context of ongoing research, is evaluated as an adjunct in non-traumatic cardiac arrest, it would likely have a beneficial role in non-traumatic hemorrhage such as PPH or gastrointestinal bleedings as well [8,13]. However, it should be noted that, despite achieving almost instant hemodynamic stability above the balloon and bleeding control below, REBOA is a temporary maneuver that requires good (and ideally preset) use of the time won by its clinical application. Few studies are available regarding appropriate occlusion times, but animal studies suggest that REBOA blockage might be survivable for up to 90 min. Thus, practicing gynecologists, anesthesiologists, and others involved in the care of bleeding patients should develop joint standards as to when REBOA could be used, and, more importantly, what to do after it has been put in place.

Acute kidney injury (KDIGO III) within days after the intervention made dialysis necessary in our case. A post-renal origin could be excluded, but several other causes of the acute kidney injury can be discussed in the order of the highest to the lowest probability, as follows: (1) a pre-renal cause such as hypovolemia during hemorrhagic shock; (2) an intrinsic drug-related cause; or (3) pre-renal decreased blood flow to the kidney due to a potentially too-high placement of the REBOA. In this case, hypovolemia seems to have been the most probable cause, as the blood loss exceeded 5000 mL and hemodynamic stability could only be achieved after about three hours. Drug-related kidney injury might also be possible, or at least be an exacerbating factor, as the woman received a variety of medications during the first day of hospitalization and several are known to have potential nephrotoxicity. Celox™, a hemostat granule containing a natural polymer extracted from shrimp cells called chitosan, has been successfully used for the treatment of PPH [14]. However, little is known about the adverse effects of the intrauterine application of Celox™, especially regarding nephrotoxicity. Furthermore, data on long-term effects, including impacts on the endometrium potentially leading to Asherman syndrome and consequently to infertility, are lacking. Another potential treatment for PPH has recently been described using recombinant factor VIIa for bleeding refractory to uterotonics [15]. Along with REBOA, these additional therapies provide more management options for severe PPH prior to hysterectomy, thus allowing for the preservation of fertility, particularly in young patients. Accidental placement of the REBOA catheter above the renal arteries may, in theory, be a third possible cause of renal insufficiency but can be excluded in this case as the balloon position was visualized during the angiography.

As mentioned above, the main advantages of REBOA are the simplicity of the technique, the almost immediate bleeding control, the reduction in blood loss, and thus the fast hemodynamic stabilization. The risks of REBOA usage are due to misplacement of the balloon, as well as ischemia and reperfusion lesions, depending on the occlusion time and scheme. Rare complications seem to be arterial thrombosis and arterial pseudoaneurysms [2,11].

Future studies should focus on the following aspects of REBOA: standardizing the use of REBOA in severe PPH; defining the ideal moment for intervention indication; explaining the best materials that should be used; defining the best localization of the balloon in the infra-renal aorta and appropriate occlusion times; and the feasibility of REBOA usage in low-resource settings as a time gain for definite treatment. Furthermore, the adverse effects of REBOA use must be analyzed in greater detail.

## 4. Conclusions

We presented the case of a young primigravida with severe postpartum hemorrhage and refractory hypovolemic shock, despite extensive surgical management and mass transfusion. A REBOA with an intra-aortic balloon placement in the infra-renal aorta achieved bleeding control and hemodynamic stability almost immediately. Our case, in combination with those in the existing literature, emphasizes the use of REBOA as a rescue strategy until definitive treatment is achieved in severe PPH. Especially in low-resource settings, such as primary and secondary centers, REBOA should be considered as a treatment strategy for severe PPH (as only relatively common skills and equipment are required) in order to gain time to organize a definite treatment or transfer to a tertiary care center. Furthermore, REBOA, in combination with transcatheter artery embolization, can potentially save women from having an emergency hysterectomy.

## Data Availability

The data presented in this study are available on request from the corresponding author. The data are not publicly available due to privacy regulations.
